# COVID-19 response measures and violence against children

**DOI:** 10.2471/BLT.20.263467

**Published:** 2020-09-01

**Authors:** Amiya Bhatia, Camilla Fabbri, Ilan Cerna-Turoff, Clare Tanton, Louise Knight, Ellen Turner, Michelle Lokot, Shelley Lees, Ben Cislaghi, Amber Peterman, Alessandra Guedes, Karen Devries

**Affiliations:** a Department of Global Health and Development, London School of Hygiene and Tropical Medicine, 15-17 Tavistock Place, Saint Pancras, London, WC1H 9SH, England.; b Department of Public Policy, University of North Carolina at Chapel Hill, Chapel Hill, United States of America.; c United Nations Children’s Fund Office of Research Innocenti, Florence, Italy.

In the early stages of the coronavirus disease 2019 (COVID-19) response, children were described as invisible carriers who posed a risk of infection to others. Here we outline how responses to COVID-19 may increase children’s exposure to violence and neglect. We also highlight ongoing efforts to address violence against children and argue for continued action and research on violence prevention within the COVID-19 response.

Globally, over 1 billion children (aged 2–17 years) experience sexual, physical or emotional violence each year.^1^ Understanding the relationship between COVID-19 and violence against children is complex, as COVID-19 measures can affect both the experience and reporting of violence. In addition, interviewing children about violence during lockdown presents ethical and methodological challenges. A review of studies on the relationship between COVID-19 and violence against women and children found 12 studies, of which only one examined violence against children and reported decreases in calls to the Child Abuse Hotline in Florida, United States of America, largely due to school closures and teachers being unable to report violence.^2^ Needs assessments from the Bolivarian Republic of Venezuela and Colombia and reported increased calls to helplines and increased risk of violence against children.^3^ Studies that rely on such violence being reported through helplines cannot detect changes in its underlying incidence or prevalence. Anecdotal evidence from previous epidemics suggests that violence against children may increase.^4^^,^^5^ During a 2017 cholera outbreak in Yemen, children with sick caregivers slept alone outside cholera treatment centres, exposed to increased risk of harassment and violence.^5^ Qualitative studies during Ebola virus outbreaks in the Democratic Republic of the Congo and Sierra Leone found children reported more frequent experiences of physical violence, and community members perceived an increased risk of violence against women and girls.^5^ The following factors were cited as reasons for the increased risk of violence: parental stress and tension, children’s increased presence at home and commercial sexual exploitation to meet economic needs.^5^

Responses to COVID-19 have included or led to restricted economic activity, school closures, reduced access to health, social and legal services, and social distancing measures. Each of these may affect the risk of violence. Increased economic insecurity could increase caregivers’ stress levels and likelihood of using violence against children and others within the household. School closures have affected over 1.5 billion children^6^ and put strain on children^7^ and caregivers’ mental health, reduced access to school-based resources, such as food and counselling, and increased the contact children have with violent caregivers. Reduced health and protection services for children further limit opportunities to identify, report and respond to violence. Finally, social distancing measures reduce child and caregiver contact with formal and informal support structures that often play a role in violence prevention and response. These pathways to violence operate across society over time and are exacerbated by unequal access to pandemic response efforts, as well as by pre-pandemic levels of health-care access, poverty, gender and social inequality.

Efforts to mitigate the effects of COVID-19 response measures on violence against children should be an essential component of pandemic response and recovery.^8^^,^^9^ Several countries have implemented policies of paid sick leave for caregivers, childcare support and child feeding programmes.^10^ Others have designated violence services as essential, relied on child helplines to receive reports of violence or have integrated child protection into COVID-19 response helplines. Efforts to respond to violence against women, which may benefit children as well, include altering stay-at-home orders for women experiencing violence, making phone helplines and smartphone apps available inconspicuously, allowing women to access shelters with their children and enabling reporting of violence via pharmacies or supermarkets.^11^ Efforts that merit further consideration include integrating child protection into existing health services, training non-traditional actors to receive disclosures of violence against children, and incorporating strategies to reduce the risk of separating children from caregivers and to promote kinship care.^8^

Prevention of violence against children should be integrated into the sectors responding to COVID-19, including health, education, social protection, law and justice. Social, economic and health policies that consider the best interests of the child, commit to preventing the social inequities that may increase risk of future violence, and focus on the vulnerabilities of displaced and refugee children, children with disabilities, and children living on the street, in detention, or in alternative care, should be central to these efforts.^12^ How stakeholders monitor and respond to violence against children during COVID-19 will have immediate and long-term implications on health, development and rights. 
